# Familiar Face Detection in 180ms

**DOI:** 10.1371/journal.pone.0136548

**Published:** 2015-08-25

**Authors:** Matteo Visconti di Oleggio Castello, M. Ida Gobbini

**Affiliations:** 1 Department of Psychological and Brain Sciences, Dartmouth College, Hanover, New Hampshire, United States of America; 2 Dipartimento di Medicina Specialistica, Diagnostica e Sperimentale (DIMES), Medical School, University of Bologna, Bologna, Italy; Monash University, AUSTRALIA

## Abstract

The visual system is tuned for rapid detection of faces, with the fastest choice saccade to a face at 100ms. Familiar faces have a more robust representation than do unfamiliar faces, and are detected faster in the absence of awareness and with reduced attentional resources. Faces of family and close friends become familiar over a protracted period involving learning the unique visual appearance, including a view-invariant representation, as well as person knowledge. We investigated the effect of personal familiarity on the earliest stages of face processing by using a saccadic-choice task to measure how fast familiar face detection can happen. Subjects made correct and reliable saccades to familiar faces when unfamiliar faces were distractors at 180ms—*very rapid saccades* that are 30 to 70ms earlier than the earliest evoked potential modulated by familiarity. By contrast, accuracy of saccades to unfamiliar faces with familiar faces as distractors did not exceed chance. Saccades to faces with object distractors were even faster (110 to 120 ms) and equivalent for familiar and unfamiliar faces, indicating that familiarity does not affect *ultra-rapid saccades*. We propose that detectors of diagnostic facial features for familiar faces develop in visual cortices through learning and allow rapid detection that precedes explicit recognition of identity.

## Introduction

As social animals we rely on interaction with conspecifics for survival. The complexity of our brains might have stemmed from the processing demands of living in groups [1–3]. The face represents one of the most efficient means to convey and receive social information. The human visual system appears to be tuned to these informative stimuli. Indeed, Crouzet and colleagues demonstrated that the fastest reliable saccade to faces occurs with an extraordinarily short latency of 100ms [4], and showed that the visual system exploits low-level visual features specific to faces—carried by the amplitude spectrum of spatial frequencies [5]—to achieve these *ultra-fast* saccades.

However, while we glance at unfamiliar faces for signs of threat or interest—during brief interactions—we spend most of our time looking at the faces of our relatives, friends, and loved ones. Behavioral evidence suggests that familiar faces have a more robust representation in the brain [6], strengthened and stabilized over the course of repeated interactions, with underlying distributed and specialized pathways for different aspects of face perception [7,8]. Moreover, familiar faces are detected faster in the absence of awareness and with reduced attentional resources [9], suggesting that our visual system is tuned to learned features of familiar faces that facilitate detection before explicit recognition of identity.

Tuning to specific features of a face is already known to occur with facial expressions [10], but may be based on stereotypical features and could be due to innate mechanisms—they are universal across cultures [11] and even produced spontaneously by the congenitally blind [12]. Perceiving faces with expressions of emotion modulates potentials even earlier than the N170 [13], but modulation of the N170 by familiarity is inconsistent across studies [14–21]. The cortical pathway for processing facial expression might be different from that for identity, and there may even be a contribution from a subcortical pathway [22–25]. By contrast, familiarity for particular faces, such as relatives and friends, must be learned and is associated with its own distributed cortical system [7,26]. The earliest and consistent modulation of neural activity evoked by faces that shows an effect of familiarity occurs long after the earliest modulation that shows an effect of facial expression. Unlike facial expressions, the diagnostic features for overlearned familiar faces are idiosyncratic (cf. caricatures).

In this experiment, we tested the effect of the familiarity of faces on the performance of a saccadic choice task. Bias toward faces could be due to innate mechanisms. Using personal familiar faces can help us understand how learning shapes the visual system for fast, optimized processing of socially relevant stimuli. This task shows faster behavioral responses than do manual response tasks [4,5,27]. We wanted to investigate how fast familiar faces can be reliably detected. Unlike perception of facial expressions, fast detection of familiar faces relies on previous learning of specific features of a face and can, therefore, reveal the effect of familiarity on early stages of visual processing. We tested the saccadic reaction time for familiar faces with either objects or unknown faces as distractors. We reasoned that because low-level visual features appear to drive *ultra-fast* saccades to faces when objects are distractors, and these features are shared between familiar and unfamiliar faces, familiarity would not affect saccadic choice reaction time on this task. By contrast, the learned visual features that distinguish personally familiar faces should affect choice saccades between familiar and unfamiliar faces.

The minimum reaction time for reliably accurate saccades to a familiar face when the distractor was an unfamiliar face was 180ms, 30–70ms earlier than the earliest evoked potential that is modulated by familiarity, the N250 [21]. We propose that these *very fast saccades* to familiar faces might be driven by detectors for diagnostic features associated with overlearned familiar faces. These detectors enable the visual system to select a familiar face before the representation of that face’s identity is activated. As predicted, familiarity did not accelerate *ultra-fast* saccades to faces when objects were distractors, which were reliably accurate at latencies shorter than 120ms, suggesting that these ultra-fast responses are driven by features that do not play a role in distinguishing familiar from unfamiliar faces.

## Methods

### Subjects

Eight subjects (two sets of four friends who knew each other for more than a year) were recruited from the Dartmouth College community and seven of them participated in the experiment (three females overall, the eighth subject did not respond for the final experiment).

Previous experiments adopted between 4 and 12 participants to study the minimum reaction times with faces [4,5]. We ran two pilot experiments with 4 participants each (these participants were not recruited for the final experiment on familiar face detection) and obtained comparable results to previous studies using the same unfamiliar faces and objects that Crouzet et al. [4] employed in their experiment.

### Ethics Statement

All subjects gave written informed consent to use their pictures as stimuli and to participate in the experiment, which was approved by the local IRB committee (IRB protocol 21200, Department of Psychological and Brain Sciences, Dartmouth College, Hanover 03755, USA). The study was conducted according to the principles of the Declaration of Helsinki.

### Stimuli

**Fig 1:**
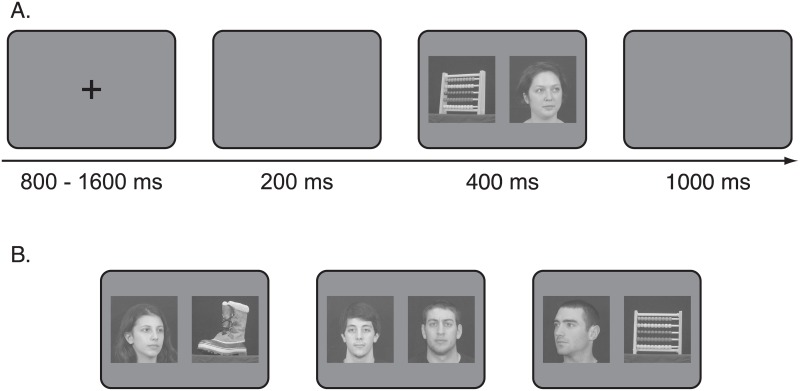
Example of the paradigm (A) with stimuli used in the experiment (B).

For each subject we took pictures of the faces of three friends with three different head orientations (frontal view with direct gaze, and head turned approximately 40 degrees to the left and to the right with averted gaze). Thus, nine pictures (three identities with three head orientations) were used as familiar-face stimuli for each subject. We used three different images for each identity (familiar and unfamiliar) in three different orientations to prevent idiosyncratic responses to a single image. The choice of the image for target and distracter was random in order to minimize expectations of identity. Pictures of students taken at a different institution, the University of Vermont, with the same setting for the familiar faces served as unknown controls. Unknown-face stimuli depicted individuals with the same head and gaze orientation as the familiar stimuli (see Fig 1 for an example). For object stimuli, we took pictures of three objects (a boot, a cup, and an abacus) in three different orientations around their main axis. Thus, each category (Unknown Faces, Familiar Faces, Objects) had three different stimulus identities in three orientations. We matched all stimuli in average luminance (expressed in pixel intensity ranging from 0 to 255) and contrast, set to 128 and 40 respectively using the function *lumMatch* in the SHINE toolbox [4,28]. Stimuli were shown on a CRT screen set at a resolution of 800x600 pixels and an 85Hz refresh rate, at a distance of 50cm. The center of each image was 8.6 degrees from the fixation cross, and each stimulus subtended 14x14 degrees [4]. The average width and height of the objects and faces were 7.93 (SD: 1.75) and 10.47 (SD: 1.04) degrees respectively. The background was set to an average gray (128 mean pixel intensity).

### Paradigm

We adopted the saccadic-choice paradigm developed by Crouzet et al. [4,27]. Each trial started with a fixation cross that lasted between 800–1600ms, then followed by a 200ms blank screen. The two stimuli then appeared for 400ms, followed by a blank screen for 1000ms [4]. Fig 1 shows the paradigm and an example of the stimuli used in the experiment.

The experiment was divided into six blocks randomized for each participant. At the beginning of each block the eye-tracker was calibrated and validated with a five-point calibration. In each block subjects had to make a saccade to stimuli belonging to one and only one category (Unknown Faces, Familiar Faces, or Objects), while distractors belonged to only one of the other categories, resulting in six target category by distractor category conditions. In half the trials the target was in the left hemifield. Each block consisted of 162 trials: we presented each possible combination of targets and distractors (9 unique target-images x 9 unique distractor-images = 81 trials) in both hemifields. Each image was shown 18 times in each block, and the order of the trials was randomized. Subjects practiced the paradigm before the experiment with a set of stimuli not used in the actual experiment (Faces and Houses). They took a small break between each block, and a longer break after the third one. Subjects performed the experiment with their chin on a chinrest. The experiment was coded in Psychtoolbox-3 [29].

### Eye movement recordings and saccade detection

Eye-movements were recorded using an Eyelink 1000 Plus with a sampling rate of 1000Hz, tracking the right eye. Saccade detection was performed offline using custom MATLAB scripts as follows. First, we detected the direction of the saccade based on the first point where the x-coordinate of the eye-position crossed an image border. Then, as potential starting points we considered those where the velocity was greater than three standard deviations of the velocity in the period before stimulus onset (blank-period, 200ms). Among these points, we then selected as the saccade onset the one where the sign of the first derivative last changed, and remained constant until the coordinate crossed the image border [30]. To control for outliers, we rejected all trials where the start of the saccade occurred before 80ms from stimulus onset [5]. We also rejected trials in which subjects failed to maintain fixation for the first 80ms of the trial.

### Statistical analyses

We performed all statistical analyses in R (version 3.1.1, [31]) using the *lme4* (version 1.1–7, [32]), *car* (version 2.0–21, [33]) and *boot* (version 1.3–11, [34]) packages and custom-made functions. Plots were made with the *ggplot2* (version 1.0.0, [35]) package.

To detect the minimum reliably accurate Saccadic Reaction Time (SRT), we followed the analyses by Crouzet et al. [4]. We divided the time from stimulus onset into 10ms time-bins (left-inclusive, e.g., the 120ms time-bin contained saccades that started between 115ms and 124ms) and looked for those in which the number of correct trials was statistically higher than the incorrect ones, using a chi-square test at p < .05 (Monte Carlo simulation, 2000 replications [36]). If we found five such consecutive time-bins, the first one was considered the minimum SRT [4].

We analyzed SRTs and accuracies using Linear Mixed-Effects and Logit Mixed-Effects Models. These models cast the problem of statistical significance into a regression framework: one tests which variables significantly contribute to predicting the dependent variable. This presented several advantages compared to a standard ANOVA. First, it allowed us to account for the subject-specific and stimulus-specific variability, by adding a random-effect structure that included both subjects and stimuli. Second, these models are more robust to designs with unbalanced data, as it might occur in a saccadic reaction-time paradigm where trials have to be rejected. Third, statistical significance of the single predictors can be tested through semi-parametric bootstrapping, which yields confidence intervals for the parameter estimates and allows one to draw inferences on the direction of the effects found. The reader is referred to Baayen, Davidson & Bates [37], Pinheiro & Bates [38], and Jaeger [39] for a detailed discussion of these models.

For both types of analyses we followed the same procedure to determine significant random and fixed effects. We first built a general model (estimated through Maximum Likelihood) and in a step-wise procedure we removed single random effects to create reduced models. Then, we tested the reduced model against the full model using a Log-likelihood Ratio Test [37,38] to determine which random effects should be kept. To test the significance of the fixed effects, we analyzed the deviance of the models using a Type III Analysis of Deviance as implemented in the package *car* [33]. Lastly, for the final models we computed confidence intervals of the parameters through parametric bootstrapping, as implemented in *lme4* [32], using 10,000 repetitions. The Linear Mixed-Effects Model was first recomputed using Restricted Maximum Likelihood Estimation, which provides better estimates of the parameters [37,38].

### Results

We rejected 5.8% of the total number of trials (6,804) according to the criteria described above. For three subjects one of the target familiar faces had a darker skin color than the other faces. We rejected all the trials containing images of this individual (both as a target and as a distractor) to avoid any bias due to a skin color difference. Thus, 5,790 trials remained for the analysis, 79.9% of which were correct. S1 Table reports the number of trials rejected for each condition. S1 and S2 Figs, and S6 and S7 Tables report data for single subjects.

### Minimum SRT

**Fig 2:**
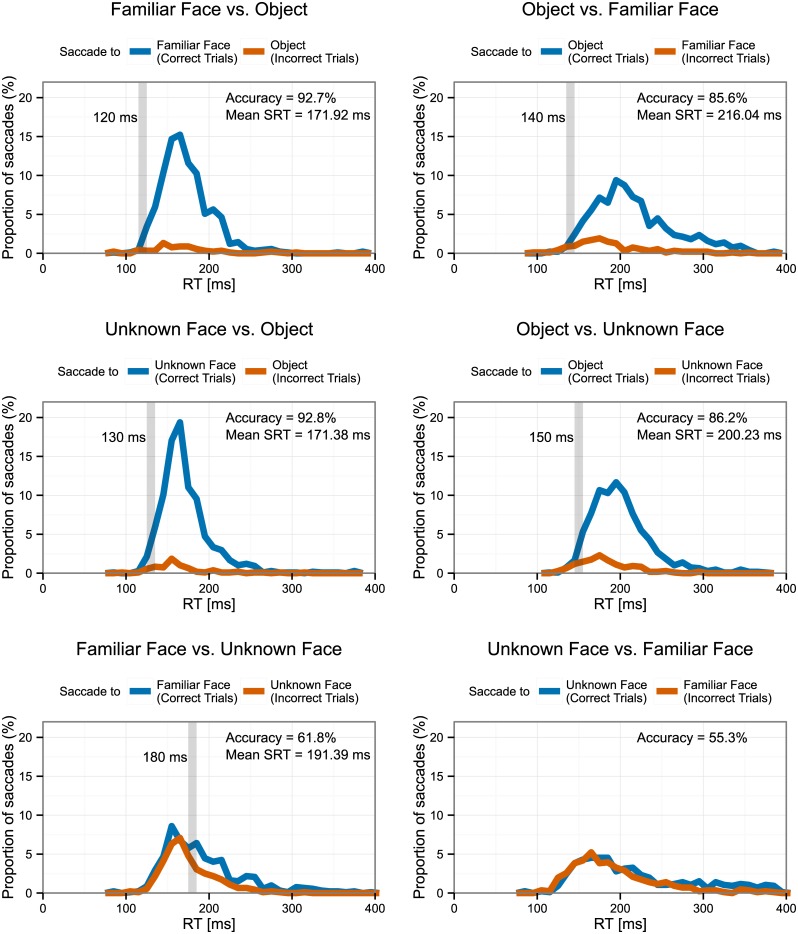
Proportion of correct (blue) and incorrect (red) saccades for each task. Gray vertical bar represents minimum SRT (see Methods for definition). Average SRTs are reported only for tasks significantly different from chance.

Fig 2 shows the proportion of correct and incorrect saccades by latency, and highlights the minimum SRT for each task (see Materials and Methods for the statistical criterion). The fastest reliably accurate saccades to familiar faces with unknown face distractors were at 180ms. No minimum SRT for Unknown Faces vs. Familiar Faces could be detected, as the overall accuracy of saccades in this task was at chance. Minimum SRTs to faces with object distractors were much faster but equivalent for familiar and unfamiliar faces (120 ms and 130 ms, respectively). Minimum SRTs to objects with face distractors were slower than saccades to faces with object distractors but faster than saccades to faces with face distractors (140 ms with familiar face distractors and 150 ms with unfamiliar face distractors).

### Mixed models on Accuracy and Reaction Times

For both type of analyses we entered the type of task, the position of the target, and their interaction as fixed effects. To account for effects due to the repetition of the images over the course of the experiment, for each subject we added a variable indicating the number of the trial (1 to 972), and entered this predictor as an additional fixed effect. After determining significance of the fixed effects with a Type 3 Analysis of Deviance, we recomputed the models without intercept to look directly at the parameter estimates and their confidence intervals for the different tasks. Moreover, we ran the analysis changing the reference level for the Target Position variable; this allowed us to obtain confidence intervals for the tasks in both hemifields.

For the analysis of accuracy, we entered each subject’s trial outcome (correct or incorrect saccade) into a Logit Mixed Model [39]. The stimuli in our design were neither fully crossed nor fully nested within subjects. To determine the best random effect structure of the model, we created two general models with Trial Number, Task, Target Position, and the interaction between Task and Target Position as fixed effects, and we varied the random effects. In the first model we entered subjects, target items, and distractor items completely crossed; in the second model we entered subjects and the combinations of subjects and target items, and of subjects and distractor items as random effects. The trial number predictor was scaled in order to avoid large eigenvalues that prevented the model to converge for both analyses on accuracy and reaction times. The two models had the same number of parameters, and thus we chose the model with higher log-likelihood, which was the first model.

Then, we tested by means of a log-likelihood ratio test [38] whether each random effect was significantly contributing to the model. We removed them one at a time and tested the reduced models against the general model described above. Each random effect was significant: subjects X2(1) = 11.88, p < .001, target items X2(1) = 7.38, p < .01, and distractor items X2(1) = 16.48, p < .001. S2 and S3 Tables show all the parameter estimates of the model computed through Maximum-Likelihood Estimation.

For the analysis of reaction times, we analyzed the saccadic RTs of correct trials only, log-transforming the data to account for the skewness of the distribution of SRTs. We then determined the random effects structure as described above for the Logit Mixed-Effects Model, and the best model in terms of higher log-likelihood was the one with subjects, the combination between subjects and target items, and between subjects and distractor items.

All three random effects were significantly contributing to the fit of the model, and were kept (subjects: X2(1) = 53.20, p < .001; subjects and target items: X2(1) = 82.79, p < .001; subjects and distractor items: X2(1) = 152.92, p < .001). S4 and S5 Tables show the parameter estimates of the model computed through Restricted Maximum-Likelihood Estimation.

### Analyses of Accuracy

**Fig 3:**
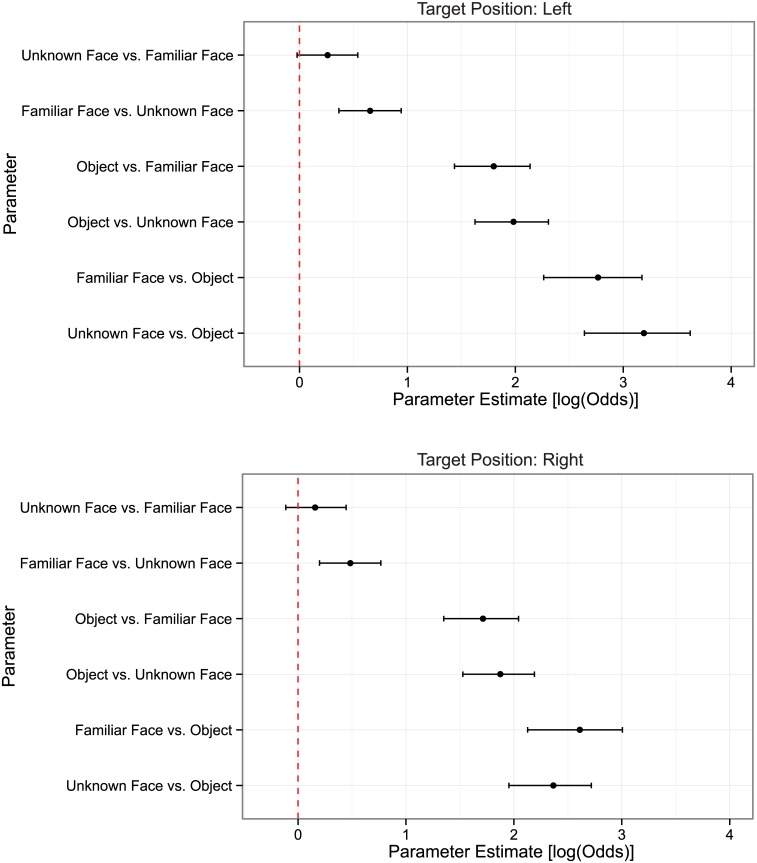
Parameter estimates for Task in the Logit Mixed-Effects Models obtained by changing reference level for target position. Error bars represent 95% bootstrapped confidence intervals.

Table 1 reports the accuracies for each condition, and Fig 3 shows the parameter estimates (log Odds) of the model for the Task variable with 95% confidence intervals computed through parametric bootstrapping with 10,000 repetitions. Since the model was computed without intercept, all parameter estimates are contrasted against 0, or chance level. We thus determined that a task was significantly above chance if its confidence interval did not contain 0. Moreover, confidence intervals can be used to judge whether parameter estimates for two tasks are likely to be different.

**Table 1:** Accuracy and mean RTs for each condition.

Task	Target Position	Accuracy [%]	SRT [ms]
**Familiar Face vs. Object**	Overall	92.72	172
	Left	93.20	172
	Right	92.22	172
**Object vs. Familiar Face**	Overall	85.59	216
	Left	86.11	216
	Right	85.07	216
**Unknown Face vs. Object**	Overall	92.77	171
	Left	95.39	170
	Right	90.13	173
**Object vs. Unknown Face**	Overall	86.21	200
	Left	86.85	206
	Right	85.58	195
**Familiar Face vs. Unknown Face**	Overall	61.81	191
	Left	63.76	190
	Right	59.87	193
**Unknown Face vs. Familiar Face**	Overall	55.28	n.s.
	Left	56.60	n.s.
	Right	54.00	n.s.

The accuracy for the Unknown Face vs. Familiar Face task did not differ significantly from chance, and thus we do not report the SRTs for that task.

We found a significant main effect of task (X2(5) = 248.69, p < .001), while the effect of the position of the target (left or right hemifield) was not significant (X2(1) = 0.53, p = .47), as well as the interaction between task and target position (X2(5) = 7.39, p = .19). The main effect of the trial number predictor was significant (X2(1) = 16.68, p < .001), with a parameter estimate of -0.16 [-0.25, -0.09], showing that accuracy decreased over the course of the experiment, although with a very small effect size, likely due to fatigue.

All tasks but the Unknown Face vs. Familiar Face were significantly above chance (see Fig 3: parameter estimates for Unknown vs. Familiar Face in left hemifield: 0.2602 [-0.0225, 0.5402]; in right hemifield: 0.1593 [-0.1337, 0.4457]). However, we found differences among the tasks. Subjects were extremely accurate when moving their eyes to a face when an object was a distractor, and their behavior was not affected by the familiarity of the face (Familiar: 92.72%, Unknown: 92.77). Moving the eyes to an object was harder and also independent of the familiarity of the face-distractor (Familiar distractor: 85.59%; Unknown distractor: 86.21%) Accuracy for saccades toward a familiar face with an unfamiliar face distractor was also significantly higher than chance (Familiar Face vs. Unknown Face: 61.81%; parameter estimate in left hemifield: 0.6550 [0.3652, 0.9423]; right hemifield: 0.4845 [0.1993, 0.7674]). S2 and S3 Tables report all the parameter estimates of the models with confidence intervals.

### Analyses of SRTs

**Fig 4:**
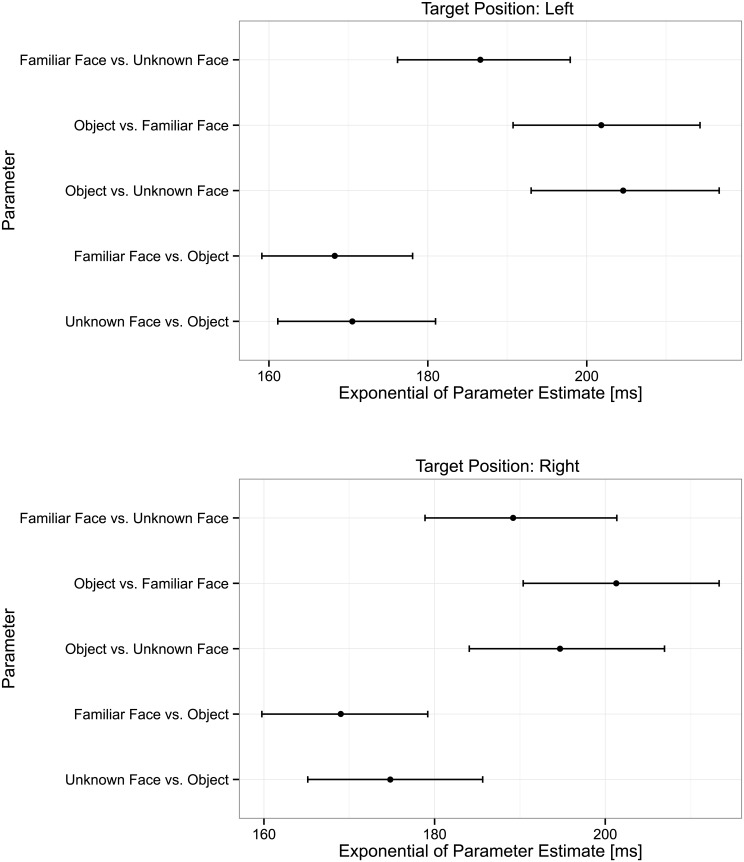
Parameter estimates for Task in the Linear Mixed-Effects Models obtained by changing reference level for target position. Error bars represent 95% bootstrapped confidence intervals.

Subjects were at chance level in the task Unknown Face vs. Familiar Face, thus we removed all trials belonging to that task before fitting the model, and considered the SRTs of correct trials only. Since we log-transformed the data to account for the skewed SRT distribution, we report the exponential of the estimates to obtain estimates in milliseconds. Fig 4 shows the exponential of parameter estimates of the model for the Task variable with 95% confidence intervals computed through parametric bootstrapping with 10,000 repetitions.

We found a significant main effect of target position (X2(1) = 6.09, p < .05) with slightly slower saccades to the right hemifield, although the effect size of the difference was small: exponential of the parameter estimate of the model for target in the right hemifield 1.0252 [1.0060, 1.0463]. The interaction between task and target position was significant (X2(4) = 29.49, p < .001), with faster saccades to the right for the task Object vs. Unknown Faces (see text below). A significant main effect of trial number (X2(1) = 179.79, p < .001) showed that, as expected, reaction times were faster later in the experiment (exponential of parameter estimate 0.9473 [0.9392, 0.9551]), and that introducing this predictor accounted for learning effects on reaction times.

Lastly, the main effect of task was significant (X2(4) = 244.43, p < .001). In both hemifields we found that familiarity did not affect the reaction times when faces were targets and objects were distractors (Unknown Face target: 171 ms; Familiar Face target: 172 ms; notice that in Fig 4 the confidence intervals for these tasks contain both parameter estimates). SRTs were statistically slower when subjects moved their eyes to an object and a face was a distractor, and also in this case familiarity did not have an effect in either hemifields (unknown distractor: 200ms; familiar distractor: 216ms; notice that in Fig 4 the confidence intervals for these tasks contain both parameter estimates). For the Familiar Face vs. Unknown Face task the SRTs (191 ms) were significantly slower than Faces vs. Objects—regardless of familiarity—in both hemifields (notice that in Fig 4 the confidence interval for this task does not contain any of the Face vs. Object parameter estimates in either hemifields), but also significantly faster than Objects vs. Faces in the left hemifield (Fig 4: the confidence interval does not contain any of the Objects vs. Faces parameter estimates only in the left hemifield). Lastly, the only significant interaction appeared with the Object vs. Unknown Face task: in this case, subjects were significantly faster when the target was in the right hemifield (left: 206ms, right: 195ms; exponential of the parameter estimate of the interaction in the right hemifield: 0.9283 [0.9041, 0.9561]). S4 and S5 Tables report all the parameter estimates of the models with confidence intervals.

## Discussion

In this paper, we investigated whether familiar faces were detected faster than unfamiliar faces in a saccadic choice task. Surprisingly, subjects made reliably correct saccades to faces of their friends in as little as 175–184ms when the distractor was a unfamiliar face, but were unable to make accurate saccades to unfamiliar faces when the distractor was a familiar face. By contrast, familiarity had no effect when the distractor was an object, with reliably correct saccades recorded in 115–134ms.

These results support the hypothesis of a multistep sequential process for face detection, perception and recognition (cf. Bruce and Young [40]). The first step is characterized by perception of the visual features that afford a distinction between faces and objects. After this initial response, activation of stored representation of visual appearance (Face Recognition Units in Bruce and Young’s model) affords finer discrimination among identities. Face Recognition Units can access identity-specific codes in the Person Identity Nodes and familiar individuals can be successfully recognized at the subsequent stage of processing.

Very fast saccades to familiar faces, with unfamiliar face distractors, are too fast to be driven by feedback from identity nodes or person knowledge or the formation of view-invariant face representations [41]. We propose that learning familiar faces involves tuning detectors to features that are specific to familiar faces—local features such as distinctive eyes or ears or a global feature for the face configuration at low spatial resolution—and operate at early stages after initial face detection, which drives ultrafast saccades, but before the activation of nodes for stored representations of familiar individuals. Thus, we are proposing a feed-forward familiar face detection process that does not require activation of view-invariant representation of a familiar face or activation of identity or personal knowledge. Indeed, some have argued that a feed-forward population coding in IT might be sufficient to support object recognition at very fast latencies, with evidence from computational models [42] and population decoding studies [43]. Such a process can be a first step to direct attention to socially-relevant familiar faces for further processing. This mechanism would also be consistent with our previous findings on detection of familiar faces that precedes conscious awareness [9]. Features used for the very earliest stage of face detection, which drive ultrafast saccades when distractors are objects, are not specific to familiar individuals. Because individual-specific detectors are learned for familiar faces but do not exist for unfamiliar faces, detection of unfamiliar faces is not facilitated at the next early stage.

Personally familiar faces are among the most highly learned and salient visual stimuli for humans. Face recognition in degraded images and across changes in viewpoint and expression is vastly superior for familiar than unfamiliar faces [6, 44]. The overlearned visual representations of familiar faces allow a more robust and rapid detection when attentional resources are limited, and even without awareness [9]. Familiarity also greatly accelerates the processing of social cues in faces [45]. Our current results suggest that the processing advantages conferred by familiarity extend to very early, but not the earliest stages of visual processing.

Crouzet et al.[4] reported that saccades to faces could be initiated as fast as 100–110ms when the distractors were objects, and our results are in line with this finding. The minimum reaction times for faces in our data were about 20ms slower than those reported by [4,5]. This could be due to differences in the paradigm—our experiment had longer blocks of trials and fewer training blocks—or to differences in the stimulus set—Crouzet et al. used pictures of faces and vehicles in natural settings, whereas ours were taken under controlled settings. The *ultra-rapid saccades* to faces reported by Crouzet et al. and replicated in this experiment may be based on low-level visual feature differences, such as the amplitude spectrum of spatial frequencies [4,5].

Such low-level features, however, cannot explain the *very rapid saccades* of familiar faces that we found at 175–184ms with unfamiliar faces as distractors. Because both targets and distractors were carefully matched faces, they shared low-level visual information. Thus, higher-level features that are diagnostic of overlearned familiar faces must drive these very rapid saccades. While learned identity-specific fragmentary features, such as the shape of the eyes, the curve of the cheeks, or the shape of the mouth, can be diagnostic for flagging a personally familiar face, fragmentary features of a stranger’s face do not distinguish that face as unfamiliar. Indeed, Hancock and colleagues showed that correct identification of unfamiliar faces is based on a holistic view [46]. The asymmetry in the features that distinguish familiar from unfamiliar faces could explain the asymmetry in performance for choice saccades to familiar versus unfamiliar faces. Whereas subjects could reliably detect familiar faces as quickly as 180ms after the face appeared, they were unable to perform the task when making a saccade to unfamiliar faces. This suggests that diagnostic single features that are learned for personally familiar faces can drive these very rapid saccades but are not available for unfamiliar faces. Features representing social cues, such as direction of attention signaled by eye gaze or head position, are analyzed faster if conveyed by a familiar face as compared to an unfamiliar face [45] supporting the hypothesis that single features may be diagnostic for quick detection of familiar faces.

Fast saccades toward familiar faces cannot be accounted by an expectancy effect. Participants in our experiment knew which friends were presented in the experiment. However, to control for a possible expectancy effect, we used the same number of familiar and unfamiliar identities with three different views of each of all six identities. On any given trial, therefore, the target could be any of nine images. Moreover, subjects became familiar with the nine images of the unknown identities over the course of the experiment, but saccades to these faces with familiar face distractors were not more accurate after controlling for trial repetition. It is unlikely, therefore, that reliably accurate and very rapid saccades to personally familiar faces with unfamiliar face distractors were driven by expectancy.

Known neurophysiological potentials do not distinguish reliably familiar from unfamiliar faces at 180ms, even though our behavioral data suggest that such a distinction must exist at this latency. A very early potential, the P100 in EEG [47] or the M110 in MEG [48], appears to differentiate faces from non-faces based on low-level visual features [48,49]. MEG studies that use multivariate decoding showed that the pattern of activity distinguishes responses to faces and non-faces as early as 90ms [50–52]. These very rapidly forming population responses probably also reflect low-level visual features and may be related to ultra-rapid saccades to faces when distractors are objects.

The earliest face-specific evoked potential—not driven by low-level features—peaks at around 170ms from stimulus onset (the N170 in EEG [13,53–55] and the M170 in MEG [48,49]. The N170 is modulated inconsistently by familiarity. Most studies report no modulation by familiarity [14–17,20,21,56,57], but others have reported that the N170 can be influenced by familiarity, even though the direction of the effect is inconsistent across studies, some reporting larger N170 evoked potentials for familiar faces [58–60] and others reporting smaller N170 evoked potentials [18,19]. On the other hand, the earliest and consistent responses that are modulated by familiarity have been detected at 210 to 250ms after stimulus onset [20,57,61,62], 30 to 70ms later than the earliest accurate saccade to a familiar face we report here. The source of these neural responses appears to be in the ventral temporal cortex. Later responses that are modulated by familiarity, with latencies greater than 300ms, appear to involve frontal and parietal areas [53,57,62]. The mismatch between the latencies of neural responses that are modulated by familiarity and of accurate saccades to familiar faces is especially surprising given that the selection and execution of the oculo-motor response might require an additional 20ms after the familiar face has been detected [27], suggesting that the neural response to a familiar face that elicits an accurate saccade may occur as early as 160ms.

In the monkey face patch system [63] population responses that encode a view-invariant representation of identity are found only in the most anterior temporal patch (AM) and peak after 300 ms [41]. Thus, saccades to a familiar face at 180ms probably precede the activation of a view-invariant representation of that face’s identity. With our data, we cannot exclude a fast feed forward route from early visual cortex to the frontal areas [64]. It is also highly unlikely that the top-down feedback from fronto-parietal areas—which play a crucial role in familiar face recognition [7,63–68]—contributes to initiating these very rapid saccades, although a subcortical pathway to these areas cannot be ruled out [69,70]. Thus, the very rapid saccades to familiar faces that we describe here cannot be attributed to known neural responses that distinguish familiar from unfamiliar faces. Moreover, our results suggest that saccades to familiar faces are initiated prior to recognition of that face’s identity.

We propose that the neural mechanism underlying both the rapid saccades and the detection of familiar faces without awareness [9] involves detectors for visual features that are specific to overlearned familiar faces. These features may be a face fragment, such as the distinctive shape of the eyes or mouth, forehead or cheeks, or a larger-scale facial configuration. Activation of these detectors may facilitate further processing, such as driving saccades, attracting attention, or breaking through inter-ocular suppression, before the features are integrated into an explicit representation of the face that is view-invariant and linked to the face’s identity. Familiar-identity-specific feature detectors may be a subset of detectors for facial attributes that also include detectors for features that are not specific to an identity, such as the distance between the eyes or the height of the forehead [71]. Thus, the potentials evoked by the activation of detectors for familiar-identity-specific features might not be distinguishable from the potentials evoked by the general-purpose detectors for non-identity-specific features.

## Conclusions

In this paper we showed that personally familiar faces are distinguished from unfamiliar face distractors at very rapid latencies in a saccadic choice task. The minimum reliably accurate saccades were at 175–184ms, 30 to 70ms before the earliest known neural response modulated by familiarity. To account for this surprising mismatch and building upon previous evidence [9,42], we propose that detectors for visual features specific to overlearned familiar faces exist in the visual cortex that afford detection of a familiar face before its explicit recognition.

## Supporting Information

S1 Fig Accuracy of each subject (colored points) in each task. The bar represents the average accuracy.
(PDF)

S2 Fig Average SRTs of each subject (colored points) in each task. The bars represent the average SRT.
(PDF)

S3 Fig Task order for each subject. (PDF)

S1 Table Rejected trials for each condition. The first Rejected column reports the number of trials rejected because the subject anticipated a saccade in the first 80ms, did not maintain fixation in the first 80ms of the trial, or failed to return to fixation before the trial started. The second Rejected column reports the number of trials rejected as in the first column, plus all the trials containing a familiar face that was darker than the other ones (one target identity for three subjects, see text for details).
(PDF)

S2 Table Parameter estimates of the fixed and random effects for the Logit Mixed-Effects Model on accuracy, Target Position: Left. Target Position has reference level: Left. Confidence intervals computed through parametric bootstrapping with 10,000 replications. The Trial variable was scaled to allow convergence of the model.
(PDF)

S3 Table Parameter estimates of the fixed and random effects for the Logit Mixed-Effects Model on accuracy, Target Position: Right. Target Position has reference level: Left. Confidence intervals computed through parametric bootstrapping with 10,000 replications. The Trial variable was scaled to allow convergence of the model.
(PDF)

S4 Table Parameter estimates of the fixed and random effects for the Linear Mixed-Effects Model on log(RT), Target Position: Left. Target Position has reference level: Left. Confidence intervals computed through parametric bootstrapping with 10,000 replications. The Trial variable was scaled to allow convergence of the model.
(PDF)

S5 Table Parameter estimates of the fixed and random effects for the Linear Mixed-Effects Model on log(RT), Target Position: Left. Target Position has reference level: Left. Confidence intervals computed through parametric bootstrapping with 10,000 replications. The Trial variable was scaled to allow convergence of the model.
(PDF)

S6 Table Accuracy for each subject in each task. (PDF)

S7 Table Average SRTs for each subject in each task. (PDF)
