# Supplementary figures and images for: Familiar Face Detection in 180ms
Source: PLoS One. 2015 Aug 25;10(8):e0136548. doi: 10.1371/journal.pone.0136548 (PMC4549263; doi:10.1371/journal.pone.0136548)

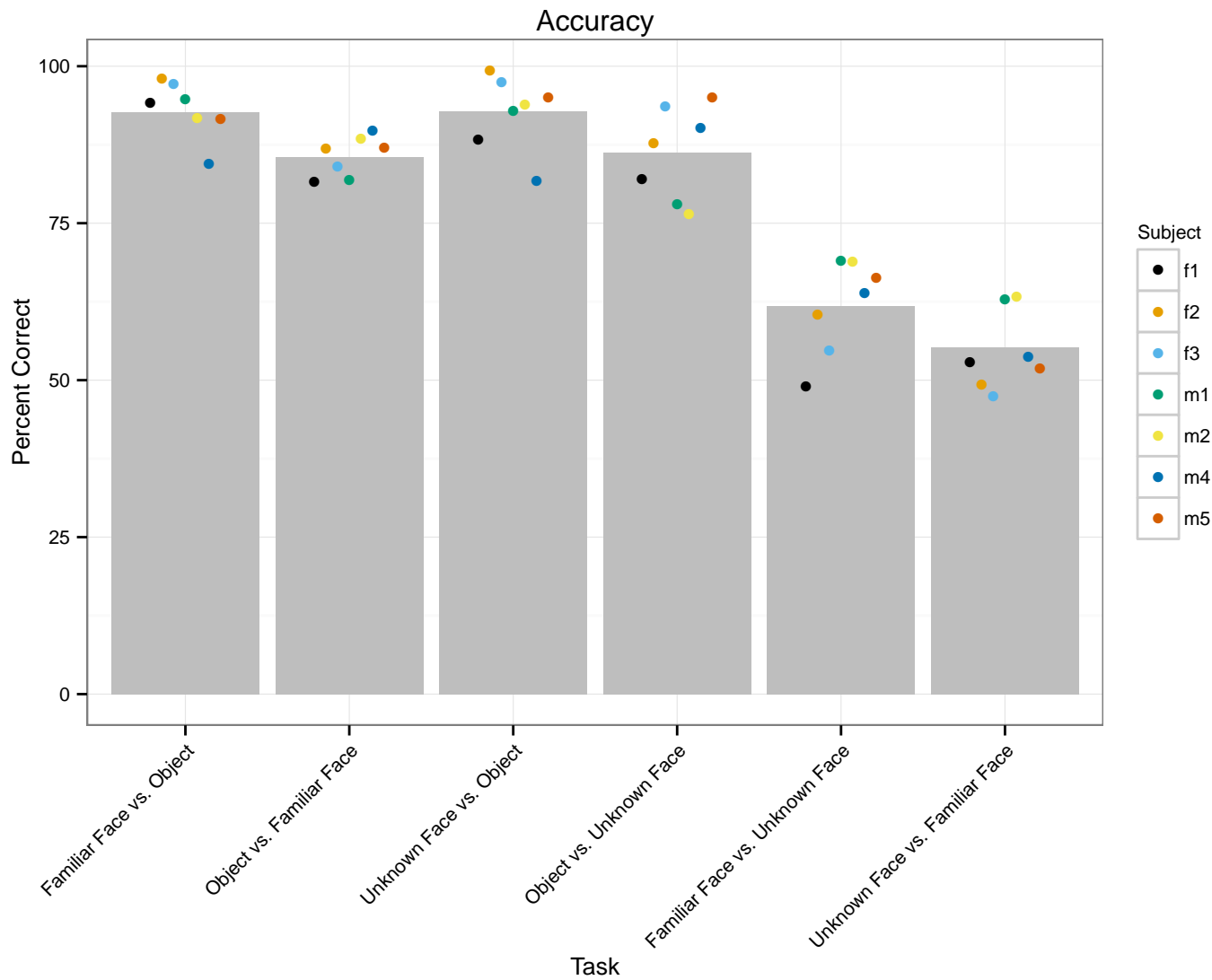

Supplement: S1 Fig — The bar represents the average accuracy. (PDF) [file pone.0136548.s001.pdf]

Average RTs

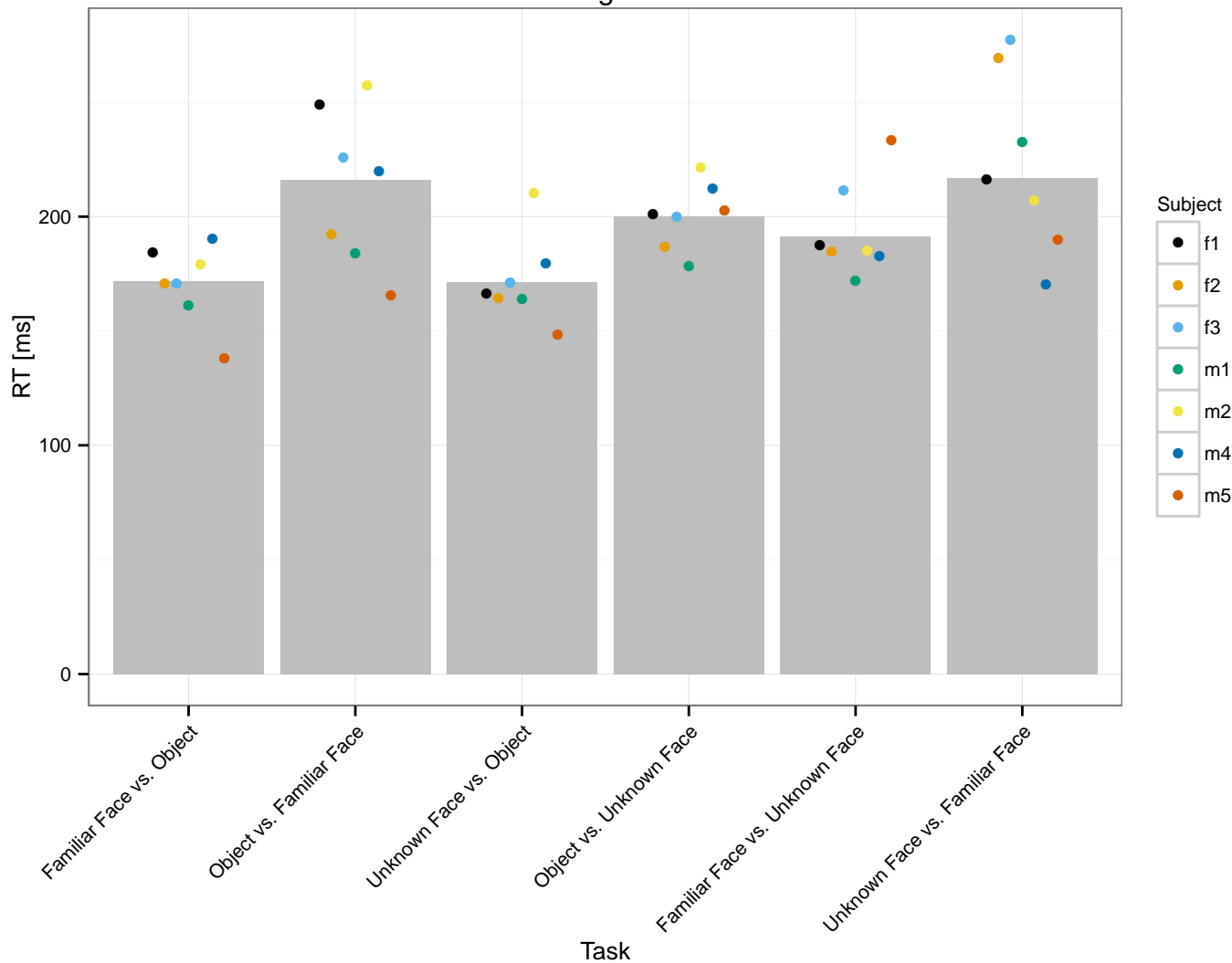

Supplement: S2 Fig — The bars represent the average SRT. (PDF) [file pone.0136548.s002.pdf]

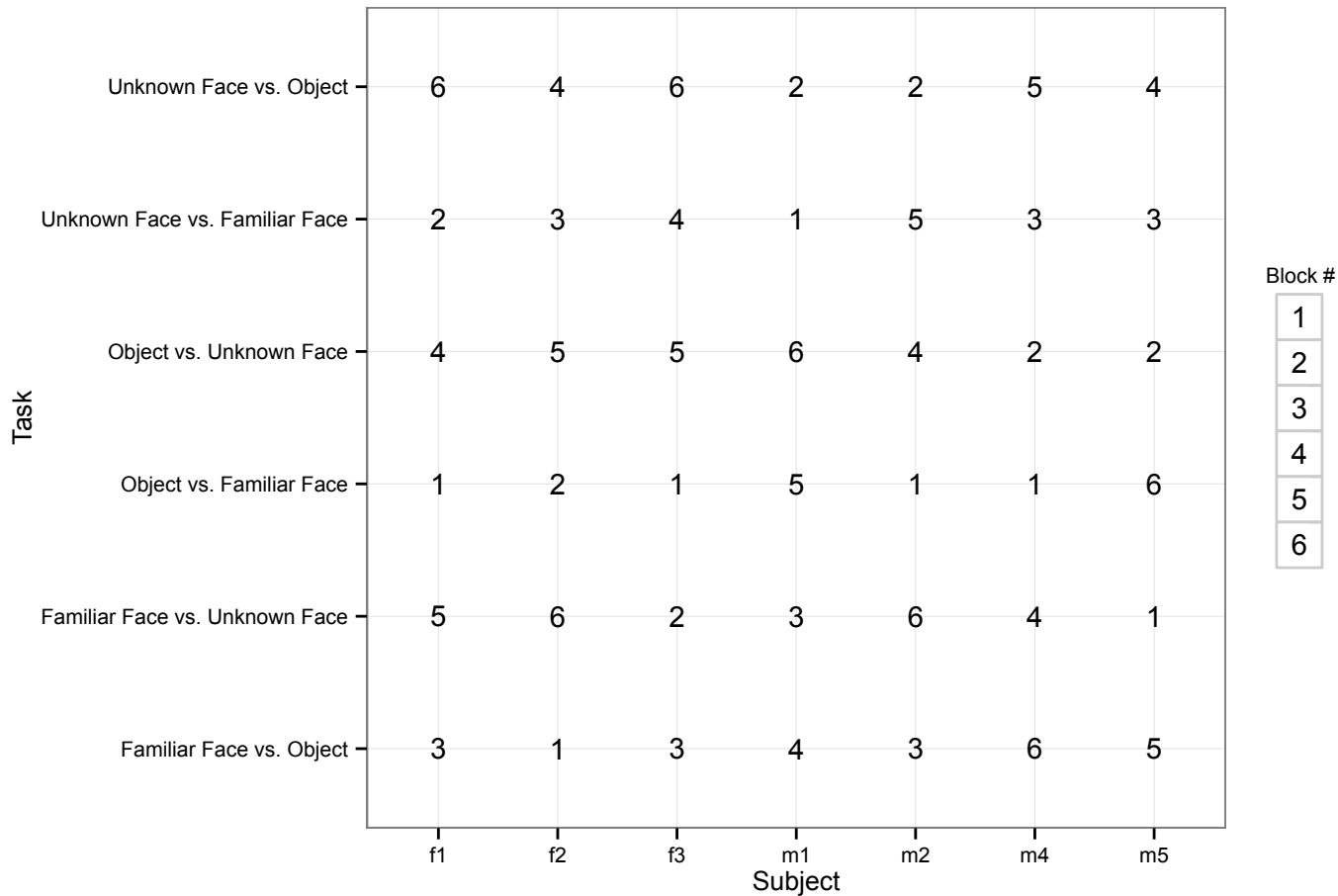

Supplement: S3 Fig — (PDF) [file pone.0136548.s003.pdf]
