# Supplementary material for: Familiar Face Detection in 180ms
Source: PLoS One. 2015 Aug 25;10(8):e0136548. doi: 10.1371/journal.pone.0136548 (PMC4549263; doi:10.1371/journal.pone.0136548)
Supplement: S1 Table — The first Rejected column reports the number of trials rejected because the subject anticipated a saccade in the first 80ms, did not maintain fixation in the first 80ms of the trial, or failed to return to fixation before the trial started. The second Rejected column reports the number of trials rejected as in the first column, plus all the trials containing a familiar face that was darker than the other ones (one target identity for three subjects, see text for details). (PDF) [file pone.0136548.s004.pdf]

Table S1. Rejected trials for each condition.

Task	Target Position	Total	Rejected	% Rejected	Rejected 2	% Rejected 2
**Familiar Face vs. Object**	Overall	1134	70	6.17	228	20.11
	Left	567	32	5.64	111	19.58
	Right	567	38	6.70	117	20.63
**Object vs. Familiar Face**	Overall	1134	38	3.35	197	17.37
	Left	567	19	3.35	99	17.46
	Right	567	19	3.35	98	17.28
**Unknown Face vs. Object**	Overall	1134	55	4.85	55	4.85
	Left	567	25	4.41	25	4.41
	Right	567	30	5.29	30	5.29
**Object vs. Unknown Face**	Overall	1134	46	4.06	46	4.06
	Left	567	27	4.76	27	4.76
	Right	567	19	3.35	19	3.35
**Familiar Face vs. Unknown Face**	Overall	1134	54	4.76	215	18.96
	Left	567	28	4.94	109	19.22
	Right	567	26	4.59	106	18.69
**Unknown Face vs. Familiar Face**	Overall	1134	134	11.82	273	24.07
	Left	567	72	12.70	143	25.22
	Right	567	62	10.93	130	22.93

Note: The first Rejected column reports the number of trials rejected because the subject anticipated a saccade in the first 80ms, did not maintain fixation in the first 80ms of the trial, or failed to return to fixation before the trial started. The second Rejected column reports the number of trials rejected as in the first column, plus all the trials containing a familiar face that was darker than the other ones (one target identity for three subjects, see text for details).
