# Supplementary material for: Familiar Face Detection in 180ms
Source: PLoS One. 2015 Aug 25;10(8):e0136548. doi: 10.1371/journal.pone.0136548 (PMC4549263; doi:10.1371/journal.pone.0136548)
Supplement: S2 Table — Target Position has reference level: Left. Confidence intervals computed through parametric bootstrapping with 10,000 replications. The Trial variable was scaled to allow convergence of the model. (PDF) [file pone.0136548.s005.pdf]

Table S2. Parameter estimates of the fixed and random effects for the Logit Mixed-Effects Model on accuracy, Target Position: Left.

Fixed Effects	log(Odds)	SE	Left CI	Right CI	Odds	Odds Left CI	Odds Right CI	Estimated Probability[a]
**Trial Number**								
Trial	-0.1651	0.0404	-0.2446	-0.0860	0.8478	0.7831	0.9176	45.88
**Task**								
Familiar Face vs. Object	2.7666	0.2261	2.2633	3.1730	15.9040	9.6151	23.8791	94.08
Familiar Face vs. Unknown Face	0.6550	0.1450	0.3652	0.9423	1.9252	1.4408	2.5659	65.81
Object vs. Familiar Face	1.7990	0.1768	1.4357	2.1371	6.0434	4.2025	8.4746	85.80
Object vs. Unknown Face	1.9831	0.1730	1.6262	2.3065	7.2649	5.0845	10.0390	87.90
Unknown Face vs. Familiar Face	0.2602	0.1443	-0.0225	0.5402	1.2972	0.9778	1.7163	56.47
Unknown Face vs. Object	3.1918	0.2424	2.6398	3.6207	24.3321	14.0106	37.3636	96.05
**Target Position**								
Right	-0.1548	0.2565	-0.6918	0.3888	0.8566	0.5007	1.4752	46.14
**Task X Target Position**								
Familiar Face vs. Unknown Face X Right	-0.0157	0.2911	-0.6150	0.5811	0.9844	0.5406	1.7880	61.88
Object vs. Familiar Face X Right	0.0694	0.3174	-0.6018	0.7131	1.0719	0.5478	2.0403	84.73
Object vs. Unknown Face X Right	0.0468	0.3113	-0.5897	0.6841	1.0479	0.5545	1.9820	86.70
Unknown Face vs. Familiar Face X Right	0.0539	0.2918	-0.5532	0.6532	1.0554	0.5751	1.9217	53.97
Unknown Face vs. Object X Right	-0.6713	0.3588	-1.3927	0.0756	0.5110	0.2484	1.0785	91.42
**Random Effects**	**sigma**	**Left CI**	**Right CI**					
**Distractor Item**	0.0779	0.0224	0.1366					
**Target Item**	0.0364	-0.0028	0.0729					
**Subjects**	0.0313	-0.0164	0.0626					

Note: *Target Position has reference level: Left. Confidence intervals computed through parametric bootstrapping with 10,000 replications. The Trial variable was scaled to allow convergence of the model.*

[a]: *The estimated probability of each task is computed as Odds/(1 + Odds), after multiplying the Odds for each task contrast.*
