# Supplementary material for: Familiar Face Detection in 180ms
Source: PLoS One. 2015 Aug 25;10(8):e0136548. doi: 10.1371/journal.pone.0136548 (PMC4549263; doi:10.1371/journal.pone.0136548)
Supplement: S3 Table — Target Position has reference level: Left. Confidence intervals computed through parametric bootstrapping with 10,000 replications. The Trial variable was scaled to allow convergence of the model. (PDF) [file pone.0136548.s006.pdf]

Table S3. Parameter estimates of the fixed and random effects for the Logit Mixed-Effects Model on accuracy, Target Position: Right.

Fixed Effects	log(Odds)	SE	Left CI	Right CI	Odds	Odds Left CI	Odds Right CI	Estimated Probability[a]
**Trial Number**								
Trial	-0.1651	0.0404	-0.2444	-0.0860	0.8478	0.7832	0.9176	45.88
**Task**								
Familiar Face vs. Object	2.6118	0.2177	2.1278	3.0054	13.6234	8.3967	20.1940	93.16
Familiar Face vs. Unknown Face	0.4845	0.1435	0.1993	0.7674	1.6234	1.2206	2.1541	61.88
Object vs. Familiar Face	1.7136	0.1736	1.3505	2.0431	5.5489	3.8594	7.7145	84.73
Object vs. Unknown Face	1.8750	0.1688	1.5267	2.1903	6.5211	4.6031	8.9380	86.70
Unknown Face vs. Familiar Face	0.1593	0.1429	-0.1137	0.4457	1.1727	0.8925	1.5616	53.97
Unknown Face vs. Object	2.3657	0.1945	1.9541	2.7179	10.6513	7.0576	15.1480	91.42
**Target Position**								
Left	0.1548	0.2565	-0.3898	0.6918	1.1674	0.6772	1.9973	53.86
**Task X Target Position**								
Familiar Face vs. Unknown Face X Left	0.0157	0.2912	-0.5789	0.6151	1.0159	0.5605	1.8499	65.81
Object vs. Familiar Face X Left	-0.0694	0.3174	-0.7131	0.6039	0.9329	0.4901	1.8293	85.80
Object vs. Unknown Face X Left	-0.0468	0.3114	-0.6841	0.5903	0.9543	0.5045	1.8046	87.90
Unknown Face vs. Familiar Face X Left	-0.0539	0.2919	-0.6576	0.5540	0.9475	0.5181	1.7402	56.47
Unknown Face vs. Object X Left	0.6713	0.3588	-0.0750	1.3929	1.9568	0.9278	4.0263	96.05
**Random Effects**	**sigma**	**Left CI**	**Right CI**					
**Distractor Item**	0.0779	0.0224	0.1366					
**Target Item**	0.0364	-0.0028	0.0729					
**Subjects**	0.0313	-0.0164	0.0626					

Note: *Target Position has reference level: Right. Confidence intervals computed through parametric bootstrapping with 10,000 replications. The Trial variable was scaled to allow convergence of the model.*

[a]: *The estimated probability of each task is computed as Odds/(1 + Odds), after multiplying the Odds for each task contrast.*
