# Supplementary material for: Familiar Face Detection in 180ms
Source: PLoS One. 2015 Aug 25;10(8):e0136548. doi: 10.1371/journal.pone.0136548 (PMC4549263; doi:10.1371/journal.pone.0136548)
Supplement: S4 Table — Target Position has reference level: Left. Confidence intervals computed through parametric bootstrapping with 10,000 replications. The Trial variable was scaled to allow convergence of the model. (PDF) [file pone.0136548.s007.pdf]

Table S4. Parameter estimates of the fixed and random effects for the Linear Mixed-Effects Model on log(RT). Target Position: Left.

Fixed Effects	Estimate	SE	Left CI	Right CI	exp(Estimate)	exp(Left CI)	exp(Right CI)	Estimated RT[a]
**Trial Number**								
Trial	-0.0542	0.0041	-0.0627	-0.0459	0.9473	0.9392	0.9551	0.95
**Task**								
Unknown Face vs. Object	5.1387	0.0283	5.0821	5.1983	170.4939	161.1140	180.9699	170.49
Familiar Face vs. Object	5.1256	0.0286	5.0696	5.1823	168.2811	159.1077	178.0845	168.28
Familiar Face vs. Unknown Face	5.2289	0.0291	5.1715	5.2879	186.5945	176.1868	197.9237	186.59
Object vs. Familiar Face	5.3074	0.0289	5.2508	5.3672	201.8315	190.7230	214.2529	201.83
Object vs. Unknown Face	5.3209	0.0284	5.2627	5.3784	204.5708	193.0027	216.6660	204.57
**Target Position**								
Right	0.0249	0.0101	0.0060	0.0453	1.0252	1.0060	1.0463	1.03
**Task X Target Position**								
Familiar Face vs. Object X Right	-0.0206	0.0150	-0.0514	0.0090	0.9796	0.9499	1.0091	169.01
Familiar Face vs. Unknown Face X Right	-0.0110	0.0170	-0.0452	0.0231	0.9890	0.9558	1.0233	189.21
Object vs. Familiar Face X Right	-0.0277	0.0152	-0.0596	0.0027	0.9727	0.9421	1.0027	201.28
Object vs. Unknown Face X Right	-0.0744	0.0145	-0.1008	-0.0449	0.9283	0.9041	0.9561	194.69
**Random Effects**	**sigma**	**Left CI**	**Right CI**					
**Distractor Item X Subject**	0.0024	0.0016	0.0032					
**Target Item X Subject**	0.0009	0.0004	0.0013					
**Subject**	0.0049	-0.0013	0.0090					

Note: *Target Position has reference level: Left. Confidence intervals computed through parametric bootstrapping with 10,000 replications. The Trial variable was scaled to allow convergence of the model.*

[a]: *Estimated RTs were computed by taking the exponential of the sum of the parameter estimates corresponding to each contrast. For Trial and Target Position, they represent a scaling factor for the parameter estimates in Task.*
