# Supplementary material for: Familiar Face Detection in 180ms
Source: PLoS One. 2015 Aug 25;10(8):e0136548. doi: 10.1371/journal.pone.0136548 (PMC4549263; doi:10.1371/journal.pone.0136548)
Supplement: S5 Table — Target Position has reference level: Left. Confidence intervals computed through parametric bootstrapping with 10,000 replications. The Trial variable was scaled to allow convergence of the model. (PDF) [file pone.0136548.s008.pdf]

Table S5. Parameter estimates of the fixed and random effects for the Linear Mixed-Effects Model on log(RT). Target Position: Right.

Fixed Effects	Estimate	SE	Left CI	Right CI	exp(Estimate)	exp(Left CI)	exp(Right CI)	Estimated RT[a]
**Trial Number**								
Trial	-0.0542	0.0041	-0.0627	-0.0459	0.9473	0.9392	0.9551	0.95
**Task**								
Unknown Face vs. Object	5.1636	0.0284	5.1067	5.2238	174.7983	165.1322	185.6348	174.80
Familiar Face vs. Object	5.1299	0.0286	5.0736	5.1885	169.0062	159.7523	179.1924	169.01
Familiar Face vs. Unknown Face	5.2428	0.0292	5.1867	5.3051	189.2077	178.8733	201.3586	189.21
Object vs. Familiar Face	5.3047	0.0288	5.2490	5.3629	201.2797	190.3804	213.3510	201.28
Object vs. Unknown Face	5.2714	0.0284	5.2153	5.3325	194.6948	184.0661	206.9465	194.69
**Target Position**								
Left	-0.0249	0.0101	-0.0453	-0.0060	0.9754	0.9557	0.9940	0.98
**Task X Target Position**								
Familiar Face vs. Object X Left	0.0206	0.0150	-0.0090	0.0514	1.0208	0.9910	1.0527	168.28
Familiar Face vs. Unknown Face X Left	0.0110	0.0170	-0.0231	0.0452	1.0111	0.9772	1.0463	186.59
Object vs. Familiar Face X Left	0.0277	0.0152	-0.0027	0.0596	1.0281	0.9973	1.0614	201.83
Object vs. Unknown Face X Left	0.0744	0.0145	0.0449	0.1008	1.0773	1.0459	1.1060	204.57
**Random Effects**	**sigma**	**Left CI**	**Right CI**					
**Distractor Item X Subject**	0.0024	0.0016	0.0032					
**Target Item X Subject**	0.0009	0.0004	0.0013					
**Subject**	0.0049	-0.0013	0.0090					

Note: *Target Position has reference level: Right. Confidence intervals computed through parametric bootstrapping with 10,000 replications. The Trial variable was scaled to allow convergence of the model.*

[a]: *Estimated RTs were computed by taking the exponential of the sum of the parameter estimates corresponding to each contrast. For Trial and Target Position, they represent a scaling factor for the parameter estimates in Task.*
