# Supplementary material for: Familiar Face Detection in 180ms
Source: PLoS One. 2015 Aug 25;10(8):e0136548. doi: 10.1371/journal.pone.0136548 (PMC4549263; doi:10.1371/journal.pone.0136548)
Supplement: S6 Table — (PDF) [file pone.0136548.s009.pdf]

Table S6. Accuracy for each subject in each task.

Task	f1	f2	f3	m1	m2	m4	m5	Overall
**Familiar Face vs. Object**	94.16	98	97.12	94.7	91.79	84.52	91.67	92.72
**Object vs. Familiar Face**	81.65	86.92	83.96	81.82	88.51	89.74	87.04	85.59
**Unknown Face vs. Object**	88.31	99.36	97.44	92.9	93.88	81.76	95.06	92.77
**Object vs. Unknown Face**	82.05	87.74	93.59	77.99	76.51	90.2	95	86.21
**Familiar Face vs. Unknown Face**	49.03	60.38	54.72	68.97	68.92	63.82	66.36	61.81
**Unknown Face vs. Familiar Face**	52.9	49.33	47.42	62.94	63.24	53.74	51.85	55.28
