# Supplementary material for: Familiar Face Detection in 180ms
Source: PLoS One. 2015 Aug 25;10(8):e0136548. doi: 10.1371/journal.pone.0136548 (PMC4549263; doi:10.1371/journal.pone.0136548)
Supplement: S7 Table — (PDF) [file pone.0136548.s010.pdf]

Table S7. Average SRTs for each subject in each task.

Task	f1	f2	f3	m1	m2	m4	m5	Overall
**Familiar Face vs. Object**	184.46	170.66	170.67	161.36	178.96	190.44	138.09	171.92
**Object vs. Familiar Face**	248.84	192.42	225.87	183.82	257.21	219.69	165.47	216.04
**Unknown Face vs. Object**	166.28	164.53	171.30	164.17	210.43	179.40	148.35	171.38
**Object vs. Unknown Face**	201.17	186.76	199.90	178.36	221.55	212.21	202.76	200.23
**Familiar Face vs. Unknown Face**	187.38	184.78	211.66	171.99	185.06	182.81	233.23	191.39
**Unknown Face vs. Familiar Face**	216.28	269.54	277.43	232.60	207.26	170.19	189.96	217.04

Note: Subjects were at chance level in the task Unknown Face vs. Familiar Face.
